# Shared genetic risk loci between Alzheimer’s disease and related dementias, Parkinson’s disease, and amyotrophic lateral sclerosis

**DOI:** 10.1186/s13195-023-01244-3

**Published:** 2023-06-16

**Authors:** Michael Wainberg, Shea J. Andrews, Shreejoy J. Tripathy

**Affiliations:** 1 Centre for Addiction and Mental Health, 250 College Street, Toronto, M5T 1R8 Canada; 2 Department of Psychiatry & Behavioral Sciences, University of California San Francisco, San Francisco, 94143 USA; 3 Institute of Medical Sciences, University of Toronto, Toronto, M5S 1A8 Canada; 4 Department of Psychiatry, University of Toronto, Toronto, M5T 1R8 Canada; 5 Department of Physiology, University of Toronto, Toronto, M5S 1A8 Canada

**Keywords:** Genome-wide association studies, Pleiotropy, Alzheimer’s disease, Parkinson’s disease, Amyotrophic lateral sclerosis

## Abstract

**Background:**

Genome-wide association studies (GWAS) have indicated moderate genetic overlap between Alzheimer’s disease (AD) and related dementias (ADRD), Parkinson’s disease (PD) and amyotrophic lateral sclerosis (ALS), neurodegenerative disorders traditionally considered etiologically distinct. However, the specific genetic variants and loci underlying this overlap remain almost entirely unknown.

**Methods:**

We leveraged state-of-the-art GWAS for ADRD, PD, and ALS. For each pair of disorders, we examined each of the GWAS hits for one disorder and tested whether they were also significant for the other disorder, applying Bonferroni correction for the number of variants tested. This approach rigorously controls the family-wise error rate for both disorders, analogously to genome-wide significance.

**Results:**

Eleven loci with GWAS hits for one disorder were also associated with one or both of the other disorders: one with all three disorders (the *MAPT*/*KANSL1* locus), five with ADRD and PD (near *LCORL*, *CLU*, *SETD1A*/*KAT8*, *WWOX*, and *GRN*), three with ADRD and ALS (near *GPX3*, *HS3ST5*/*HDAC2*/*MARCKS*, and *TSPOAP1*), and two with PD and ALS (near *GAK*/*TMEM175* and *NEK1*). Two of these loci (*LCORL* and *NEK1*) were associated with an increased risk of one disorder but decreased risk of another. Colocalization analysis supported a shared causal variant between ADRD and PD at the *CLU*, *WWOX*, and *LCORL* loci, between ADRD and ALS at the *TSPOAP1* locus, and between PD and ALS at the *NEK1* and *GAK*/*TMEM175* loci. To address the concern that ADRD is an imperfect proxy for AD and that the ADRD and PD GWAS have overlapping participants (nearly all of which are from the UK Biobank), we confirmed that all our ADRD associations had nearly identical odds ratios in an AD GWAS that excluded the UK Biobank, and all but one remained nominally significant (*p* < 0.05) for AD.

**Conclusions:**

In one of the most comprehensive investigations to date of pleiotropy between neurodegenerative disorders, we identify eleven genetic risk loci shared among ADRD, PD, and ALS. These loci support lysosomal/autophagic dysfunction (*GAK*/*TMEM175*, *GRN*, *KANSL1*), neuroinflammation/immunity (*TSPOAP1*), oxidative stress (*GPX3*, *KANSL1*), and the DNA damage response (*NEK1*) as transdiagnostic processes underlying multiple neurodegenerative disorders.

## Introduction

Neurodegenerative disorders like Alzheimer’s disease (AD), Parkinson’s disease (PD), and amyotrophic lateral sclerosis (ALS) have traditionally been viewed as clinicopathologically distinct entities. However, it is increasingly appreciated that these seemingly disparate disorders do share at least some core underlying processes. Most notably, neurodegenerative disorders almost universally involve the formation of protein aggregates (proteinopathy) in the brain regions undergoing neurodegeneration. While neurodegenerative disorders are often distinguished by their dominant types of protein aggregates—such as amyloid-beta and tau in AD, alpha-synuclein in PD and other synucleinopathies, and TAR DNA-binding protein 43 (TDP-43) in ALS [1]—patients with one neurodegenerative disorder often present with protein aggregates characteristic of another disorder [2, 3]. This is further supported by the unsupervised clustering of neurodegenerative disorder patients, which yields transdiagnostic clusters of pathology that cut across diagnostic boundaries [4]. Neurodegenerative disorders are also associated with many of the same disturbances at the molecular level, such as neuroinflammation, oxidative stress, metabolic dysregulation, mitochondrial dysfunction, and deficits in protein quality control and degradation [5–9].

This overlap at the pathological and molecular levels is also reflected at the genetic level. Most pairs of neurodegenerative disorders for which genome-wide association studies (GWAS) have been conducted do show at least some degree of genetic correlation [10], a genome-wide measure of genetic overlap, though much less than psychiatric disorders [11]. Yet, it has proven difficult to identify which specific genetic variants or loci are responsible for this overlap. The human leukocyte antigen (HLA) region, which encodes key genes involved in the adaptive immune system, was first associated with PD in 2010 [12], AD in 2013 [13], frontotemporal dementia (FTD) in 2014 [14], and ALS in 2021 [15]. The *MAPT* (tau) locus was first associated with PD in 2009 [16], AD in 2015 [17, 18], and progressive supranuclear palsy (PSP) in 2018 [19]. The *TMEM106B* locus was the first-discovered genetic risk factor for FTD with TDP-43 inclusions in 2010 [20] and was subsequently shown in 2021 to also be a risk factor for AD [21]. A recent GWAS for AD and related dementias (ADRD) [22], which pooled AD cases with “proxy cases” having a family history of all-cause dementia, used colocalization analysis to provide evidence that ADRD and FTD with TDP-43 inclusions share causal variants at the *TMEM106B* and *GRN* loci. The *GRN* locus was also shown to be associated with all three of AD, PD, and ALS in a separate study [23]. Similarly, a recent ALS GWAS [15] found colocalization with PSP and corticobasal degeneration (CBD) at the *MOBP*/*RPSA* locus, with FTD at the *UNC13A* locus, with PD at the HLA and *GAK* loci, with AD at the *TSPOAP1* locus, and with the motor neuron disease subtype of FTD at the *C9orf72* locus. Other studies have used the conjunctional false discovery rate [24] to nominate variants associated with multiple neurodegenerative disorders. Using this approach, certain FTD-associated variants have been associated with AD or PD [25], certain CBD-associated variants with PSP or FTD [26], and certain ALS-associated variants with PSP, FTD, or FTD with TDP-43 inclusions [27]. However, GWAS approaches based on false discovery rates are much less stringent than genome-wide significance/Bonferroni correction, since they do not control the family-wise error rate [28]. For instance, an FDR threshold of 0.05 implies that up to 5% of associations may be false positives, whereas a Bonferroni threshold of 0.05 implies a < 5% chance of even a single false positive.

Here, we leverage state-of-the-art GWAS for ADRD [22], PD [29], and ALS [15], three of the neurodegenerative disorders with the best-powered GWAS, to identify loci associated with multiple of these three disorders. To address the concern that ADRD is an imperfect proxy for AD, we replicate each ADRD association in an AD GWAS [30] as a sensitivity analysis. Our strategy is extremely simple: for each pair of disorders, we ask whether genome-wide significant variants for one disorder are also significantly associated with the other disorder, after applying Bonferroni correction for the number of genome-wide significant variants tested. This approach ensures rigorous control of the family-wise error rate for association with both disorders. For each shared locus we discover, we use colocalization analyses to explore whether the disorders are more likely to share a causal variant at the locus, to have distinct causal variants, or to not have a causal variant, and perform a detailed literature review to explore which causal gene(s) may mediate each locus’s association with neurodegenerative disease.

## Methods

### Genome-wide association studies

Our analysis is based on (a) independent genome-wide significant variants (“lead variants”) and (b) genome-wide summary statistics from ADRD, PD, and ALS genome-wide association studies (GWAS).

The ADRD GWAS [22] included 788,989 European ancestry participants, comprising 64,498 AD cases, 46,828 “proxy cases” from the UK Biobank [31] with a family history of dementia, and 677,663 controls. It discovered 83 lead variants at 75 risk loci. We obtained summary statistics for a subset of 487,511 participants (39,106 cases, 46,828 proxy cases, 401,577 controls) from the NHGRI-EBI GWAS Catalog [32] (https://ebi.ac.uk/gwas/studies/GCST90027158).

The PD GWAS [29] included 1,474,097 European ancestry participants, comprising 37,688 cases, 18,618 proxy cases from the UK Biobank with a family history of PD, and 1,417,791 controls. It discovered 90 lead variants at 78 risk loci. We obtained summary statistics for all participants from 23andMe, Inc. (https://research.23andme.com/dataset-access).

The ALS GWAS [15] included 138,086 European ancestry participants (27,205 cases, 110,881 controls) and 14,182 East Asian ancestry participants (2407 cases, 11,775 controls). It discovered 15 lead variants at 15 risk loci. We obtained summary statistics for all participants from the GWAS Catalog (https://ebi.ac.uk/gwas/studies/GCST90027163).

As a sensitivity analysis, we also analyzed summary statistics for AD [30] (https://ebi.ac.uk/gwas/studies/GCST007511), based on 63,926 participants (21,982 cases, 41,944 controls), alongside those for ADRD, PD, and ALS.

For genetic correlation analyses (see the next section), we used ALS summary statistics for only the 138,086 European ancestry participants (https://ebi.ac.uk/gwas/studies/GCST90027164).

### Genetic correlation

As a prelude to our primary analysis, we assessed the global genetic similarity between AD/ADRD, PD, and ALS using three genetic correlation methods: LDSC [33] (linkage disequilibrium [LD] score regression; https://github.com/bulik/ldsc), HDL [34] (high-definition likelihood; https://github.com/zhenin/HDL), and GNOVA [35] (genetic covariance analyzer; https://github.com/qlu-lab/GNOVA-2.0).

For LDSC and GNOVA, we computed LD scores using the European subset of 1000 Genomes Phase 3 [36] (https://storage.googleapis.com/broad-alkesgroup-public/LDSCORE/1000G_Phase3_plinkfiles.tgz). For HDL, we used the “1,029,876 QCed UK Biobank imputed HapMap3 SNPs” file from https://github.com/zhenin/HDL/wiki/Reference-panels as the LD matrix.

For GNOVA, we used the genetic correlation estimate with sample overlap correction (“corr_corrected” column of the GNOVA output) and the *p*-value for genetic covariance with sample overlap correction (“pvalue_corrected”). We back-calculated standard errors (used to calculate 95% confidence intervals) from this genetic correlation and *p*-value.

Prior to computing genetic correlations, we harmonized the alleles of each trait’s summary statistics to the European subset of 1000 Genomes Phase 3 using ldsc’s munge_sumstats.py script, providing an effective sample size (N_eff_) corrected for case–control imbalance instead of the true sample size. For ADRD and PD, we inferred *N*_eff_ for each variant using the formula *N*_eff_ = ((4/(2 × MAF × (1 − MAF) × INFO)) − BETA^2^)/SE^2^, where MAF is the variant’s minor allele frequency in the GWAS sample, INFO its imputation information score, BETA its effect size, and SE the standard error of this effect size [37]; since neither study provided INFO scores, we set INFO to 1. For AD, neither minor allele frequencies nor per-variant sample sizes were provided, so we inferred *N*_eff_ via the formula *N*_eff_ = 4/(1/*N*_cas_ + 1/*N*_con_), where *N*_cas_ = 21,982 and *N*_con_ = 41,944 are the total numbers of cases and controls in the GWAS. For ALS, *N*_eff_ was already provided for each variant.

### Shared genetic risk loci

For our primary analysis, we sought to find variants associated with multiple ADRD, PD, and ALS. We began with the lead variants reported by each study: 83 for ADRD, 90 for PD, and 15 for ALS. We also included rs429358, which differentiates APOE4 from APOE3, as an 84th lead variant for ADRD, since the ADRD GWAS excluded the *APOE* region. We excluded one lead variant from each study due to being in the HLA region (rs6605556 for ADRD, rs112485576 for PD, rs9275477 for ALS). This led to 83 variants for ADRD, 89 for PD, and 14 for ALS.

We pooled the lead variants for each pair of disorders, leading to 83 + 89 = 172 variants for ADRD and PD, 97 for ADRD and ALS, and 103 for PD and ALS. We then subset to only those that were also present or had linkage disequilibrium (LD) proxies, in the other disorder’s summary statistics. For instance, for the ADRD and PD analysis, we only kept ADRD lead variants that were also present (or had LD proxies) in the PD summary statistics, and PD lead variants that were also present (or had LD proxies) in the ADRD summary statistics. We used the TopLD resource [38] (https://topld.genetics.unc.edu), based on the Trans-Omics for Precision Medicine (TOPMed) whole-genome sequencing cohort [39], to define LD proxies. For each lead variant missing from the other disorder’s summary statistics, we enumerated all LD proxies with *r*^2^ > 0.8 in the European subset of TOPMed that were not missing from the summary statistics. If any were available, we selected the highest *r*^2^ one. One hundred sixty-two of the 172 lead variants for ADRD and PD were present or had LD proxies in the other disorder’s summary statistics, 88 of 97 for ADRD and ALS, and 98 of 103 for PD and ALS.

Finally, we looked up the *p*-values of these lead variants/proxies in the other disorder’s summary statistics and tabulated which were significant at a family-wise error rate of 5%, after Bonferroni correction for the number of lead variants/proxies. Thus, we used significance thresholds of *p* < 0.05/162 ≈ 3.1 × 10^−4^ for ADRD and PD, *p* < 0.05/88 ≈ 5.7 × 10^−4^ for ADRD and ALS, and *p* < 0.05/98 ≈ 5.1 × 10^−4^ for PD and ALS.

Our approach, a form of stepwise gatekeeper hypothesis testing [40], rigorously controls the family-wise error rate. This approach was also used by a 2015 analysis focused on genome-wide significant variants for PD that associated the *MAPT* locus with AD [17]. The elegance of this approach is that, by focusing variant discovery for the second disorder on only variants that are genome-wide significant for the first disorder, rather than the entire genome, it reduces the multiplicity of tests that need to be corrected for by roughly four orders of magnitude (from ~ 1 million to ~ 100). This maintains rigorous family-wise error rate control, the gold standard in statistical genetics, while enabling the association of variants with both disorders even if only genome-wide significant for one disorder.

### Colocalization analysis

Colocalization tests like coloc [41] (https://chr1swallace.github.io/coloc) estimate whether two traits share a causal variant at a locus. Given the GWAS effect sizes for each disorder for all variants at a locus, coloc calculates a posterior probability that the two disorders share the same causal variant at the locus (which coloc refers to as PP_H4_, and we refer to as PP_shared_), the posterior probability that the two disorders have distinct causal variants at the locus (which coloc refers to as PP_H3_, and we refer to as PP_distinct_), and the posterior probabilities that one or both of the disorders do not have a causal variant at the locus.

Before using coloc, we harmonized each pair of the AD, PD, and ALS summary statistics with each other to make allele codings consistent, removing ambiguous variants (A/T, C/G). For each locus, we ran coloc (specifically, the https://chr1swallace.github.io/coloc/reference/coloc.abf.html function from version 5.1.1 of the coloc package with default parameters) on all of the harmonized variants in the same approximately independent linkage disequilibrium block [42] as the lead variant. These linkage disequilibrium blocks were derived from the European subset of 1000 Genomes and are available at https://bitbucket.org/nygcresearch/ldetect-data/src/master/EUR/fourier_ls-all.bed.

## Results

### Genetic correlations among neurodegenerative disorders

**Fig. 1:**
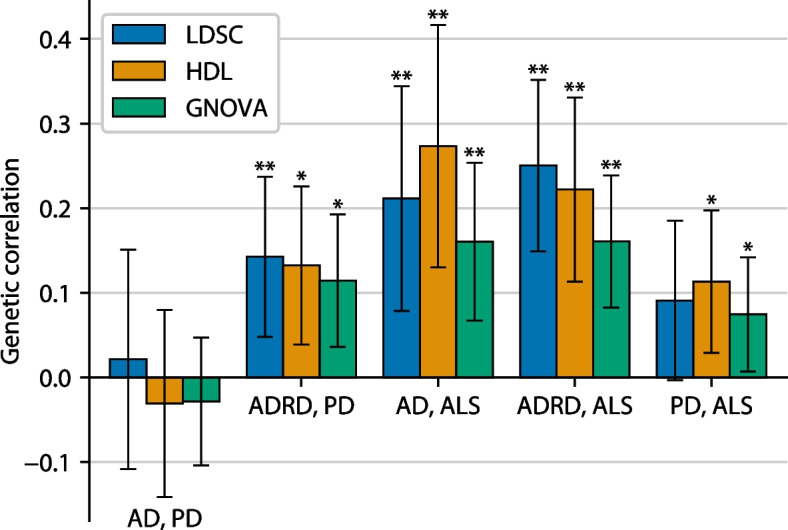
Genetic correlations between AD/ADRD, PD, and ALS according to three genetic correlation methods. Error bars indicate 95% confidence intervals. Single asterisks (*) indicate nominal significant genetic correlations (*p* < 0.05), while double asterisks (**) indicate genetic correlations significant after Bonferroni correction (*p* < 0.05/15). Note that ADRD and PD are slightly genetically correlated, while AD and PD are not, most likely due to the inclusion of non-AD dementias in the ADRD case definition (see the “Discussion” section)

Genetic correlations between AD/ADRD, PD, and ALS were generally small but positive according to all three methods tested [33–35] (LDSC, HDL, and GNOVA; Fig. 1, Table 1). The one exception was AD and PD, which had near-zero genetic correlation according to all three methods, with point estimates ranging from − 0.03 to 0.02. (Genetic correlations, like other types of correlation, generally range from −1 to +1, although values outside this range are occasionally observed.) Our genetic correlations are highly concordant with those calculated by a recent study [43] using LDSC (Table 1).

**Table 1:** Genetic correlations between AD/ADRD, PD, and ALS according to three genetic correlation methods. Tabular version of Fig. 1. Square brackets indicate 95% confidence intervals. For comparison, genetic correlations calculated from a previous study [43] are also shown. Wingo et al. used an older ADRD GWAS (Jansen et al. 2019 [44] instead of Bellenguez et al. 2022 [22]) and used a version of the summary statistics from Nalls et al. 2019 [29]  that did not include 23andMe participants

	**LDSC**	**HDL**	**GNOVA**	**Wingo et al. 2022 (LDSC)**
**AD, PD**	0.02 [−0.11, 0.15] (*p* = 0.75)	−0.03 [−0.14, 0.08] (*p* = 0.58)	−0.03 [−0.10, 0.05] (*p* = 0.46)	0.07 [−0.09, 0.23] (*p* = 0.35)
**ADRD, PD**	0.14 [0.05, 0.24] (*p* = 0.0032)	0.13 [0.04, 0.23] (*p* = 0.0054)	0.11 [0.04, 0.19] (*p* = 0.0042)	0.16 [0.04, 0.28] (*p* = 0.0055)
**AD, ALS**	0.21 [0.08, 0.34] (*p* = 0.0018)	0.27 [0.13, 0.42] (*p* = 1.8 × 10^−4^)	0.16 [0.07, 0.25] (*p* = 7.5 × 10^−4^)	0.17 [−0.07, 0.41] (*p* = 0.17)
**ADRD, ALS**	0.25 [0.15, 0.35] (*p* = 1.3 × 10^−6^)	0.22 [0.11, 0.33] (*p* = 6.3 × 10^−5^)	0.16 [0.08, 0.24] (*p* = 5.5 × 10^−5^)	0.21 [−0.01, 0.43] (*p* = 0.046)
**PD, ALS**	0.09 [−0.00, 0.19] (*p* = 0.059)	0.11 [0.03, 0.20] (*p* = 0.0085)	0.07 [0.01, 0.14] (*p* = 0.031)	0.06 [−0.12, 0.24] (*p* = 0.52)

On the other hand, ADRD and PD were genetically correlated, with point estimates ranging from 0.11 to 0.14 across the three methods. This suggests that other types of dementia besides AD may be driving the genetic correlation, perhaps especially dementia with Lewy bodies, which like PD is a synucleinopathy. Even though the ADRD and PD GWAS include overlapping samples from the UK Biobank, the genetic correlation according to GNOVA (0.11), which accounts for sample overlap, was only marginally smaller than according to LDSC (0.14) or HDL (0.13).

AD/ADRD and ALS had the highest genetic correlations of all pairs of disorders tested. Point estimates ranged from 0.16 to 0.27 for AD and ALS across the three methods, and from 0.16 to 0.25 for ADRD and ALS. Finally, PD and ALS had a slight positive genetic correlation, with point estimates ranging from 0.07 to 0.11.

### Genetic risk loci shared between ADRD and PD

**Fig. 2:**
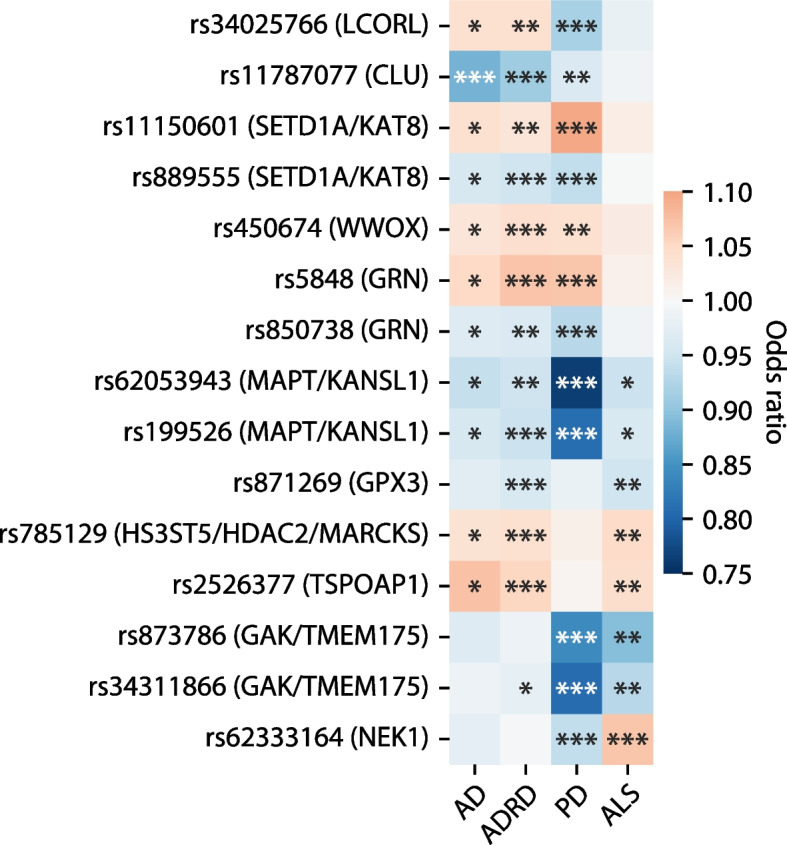
Summary of the shared loci between AD/ADRD, PD, and ALS. Variants are listed in the same order as they are presented in Tables 2, 3, and 4 (except rs199515 at the *MAPT*/*KANSL1* locus, which is not available in the PD GWAS; its LD proxy rs199526 is shown). Single asterisks (*) indicate nominal significance (*p* < 0.05), double asterisks (**) indicate significance after Bonferroni correction for the number of tested variants (see the “Methods” section), and triple asterisks (***) indicate genome-wide significance. See Tables 2, 3, and 4 for additional details

Nine lead variants at six loci were associated with both ADRD and PD at a family-wise error rate of 5%, after Bonferroni correction for the number of lead variants for both disorders (Table 2, Fig. 2). To address the concern that ADRD is an imperfect proxy for AD, we confirmed that all nine variants were nominally significant (*p* < 0.05) for AD, with nearly identical odds ratios to ADRD.

**Table 2:** Lead variants for ADRD or PD associated with both disorders at a family-wise error rate of 5%. Odds ratios and *p*-values for AD (from Kunkle et al.) are also included as a sensitivity analysis. A1 = effect allele; A2 = non-effect allele; OR = odds ratio; PP_shared/distinct_ = posterior probability that the two disorders share the same causal variant/have two distinct causal variants at the locus according to colocalization analysis. rs199526 was chosen as an LD proxy (*r*^2^ = 0.911 in TopLD) of the true ADRD lead variant, rs199515, which was not available in the PD summary statistics. For ADRD, odds ratios and *p*-values are from the full 788,989 participants where available (i.e., for ADRD lead variants, aside from the LD proxy rs199526) and from the 487,511 participants with genome-wide summary statistics otherwise (i.e., for the PD lead variants and rs199526)

Locus	Variant	A1/A2	Lead for	AD		ADRD		PD		PP_shared_	PP_distinct_	Nearest gene	Most evidence for
				**OR**	***p*-value**	**OR**	***p*-value**	**OR**	***p*-value**				
1	rs34025766	A/T	PD	1.04	0.04	1.04	2.5 × 10^−4^	0.98	2.9 × 10^−10^	65.3%	16.6%	*LCORL*	Unclear
2	rs11787077	T/C	ADRD	0.88	2.5 × 10^−16^	0.91	1.7 × 10^−44^	0.96	1.7 × 10^−4^	88.4%	1.8%	*CLU*	*CLU*
3	rs11150601	A/G	PD	1.04	0.0067	1.03	1.5 × 10^−4^	1.09	5.1 × 10^−20^	0.0%	100.0%	*SETD1A*	*SETD1A*, *KAT8*
3	rs889555	T/C	ADRD	0.96	0.0056	0.95	2.0 × 10^−11^	0.94	5.6 × 10^−9^	0.0%	100.0%	*BCKDK*	*SETD1A, KAT8*
4	rs450674	T/C	ADRD	1.03	0.038	1.04	3.2 × 10^−8^	1.04	2.1 × 10^−4^	80.5%	9.8%	*MAF*	*WWOX*
5	rs5848	T/C	ADRD	1.05	0.0029	1.07	2.4 × 10^−20^	1.07	1.8 × 10^−12^	25.8%	74.2%	*GRN*	*GRN*
5	rs850738	A/G	PD	0.97	0.035	0.96	3.0 × 10^−6^	0.93	1.3 × 10^−11^	25.8%	74.2%	*FAM171A2*	*GRN*
6	rs62053943	T/C	PD	0.94	0.0097	0.95	5.1 × 10^−6^	0.76	3.6 × 10^−68^	18.4%	81.6%	*LINC02210-CRHR1*	*MAPT*, *KANSL1*
6	rs199526	C/G	ADRD	0.96	0.014	0.95	1.7 × 10^−8^	0.81	1.8 × 10^−65^	18.4%	81.6%	*WNT3*	*MAPT, KANSL1*

The first variant associated with both ADRD and PD was the PD lead variant rs34025766 (chr4:17,967,188 in hg38 coordinates), located in an intron of the transcription factor *LCORL*. rs34025766 had opposite directions of association with ADRD (odds ratio [OR] = 1.04, *p* = 2.5 × 10^−4^) and PD (OR = 0.98, *p* = 2.9 × 10^−10^). Though an ambiguous variant (A/T), its top non-ambiguous LD partner in TopLD (see the “Methods” section), rs16896101, showed the same discordance between ADRD and PD, indicating that the discordance was not due to an allele coding issue. Colocalization analysis suggested that ADRD and PD were fairly likely (65.3% chance) to share a causal variant at this locus, and unlikely (16.6% chance) to have two distinct causal variants; note that these probabilities do not add up to 100% because the colocalization test also considers the possibility of one or both disorders having no causal variants at the locus. This suggests that the same causal variant has opposite directions of effect on the two disorders. It is unclear which gene(s) are causal at this locus.

The second variant associated with both ADRD and PD was the ADRD lead variant rs11787077 (chr8:27,607,795), located in an intron of *CLU*. Its PD association was weaker than, but concordant with, its ADRD association (ADRD OR = 0.91, *p* = 1.7 × 10^−44^; PD OR = 0.96, *p* = 1.7 × 10^−4^); colocalization analysis supported a shared causal variant (88.4% chance). *CLU* (clusterin), also called *APOJ*, is one of the earliest-discovered and strongest genetic risk factors for late-onset AD and has been proposed to affect AD risk by regulating diverse cellular processes including lipid transport, innate immunity, apoptosis, oxidative and proteostatic stress responses, and even copper homeostasis [45]. Independent of the common-variant signal at this locus, rare protein-altering variants in *CLU* have also been associated with AD [46]. While a role for *CLU* in PD has not been definitively established, extracellular clusterin was recently shown to regulate astrocytic uptake of alpha-synuclein fibrils [47], supporting its relevance to PD.

The next two variants associated with both ADRD and PD were the PD lead variant rs11150601 (chr16:30,966,478) and the ADRD lead variant rs889555 (chr16:31,111,250) approximately 150 kilobases away. Both variants have the same direction of association with ADRD and PD: rs11150601’s is associated with increased risk of both disorders, but especially PD (ADRD OR = 1.03, *p* = 1.5 × 10^−4^; PD OR = 1.09, *p* = 5.1 × 10^−20^), while rs2884738 is associated with decreased risk of both disorders, to about the same degree (ADRD OR = 0.95, *p* = 2.0 × 10^−11^; PD OR = 0.94, *p* = 5.6 × 10^−9^). Colocalization analysis strongly supports distinct causal variants for the two disorders (100.0% chance). rs11150601 lies within an intron of *SETD1A*; rs889555 lies within an intron of *BCKDK*, but its locus is typically called the *KAT8* locus by PD GWAS (*KAT8* is the second-nearest gene). Both SETD1A and KAT8 are chromatin remodelers, and both cause autosomal dominant neurodevelopmental disorders [48, 49]. KAT8 is a key regulator of autophagy [50], and the presence of a GWAS hit near *KAT8* has been cited as evidence in support of PD being a “lysosomal disorder” [51]. KAT8 also regulates *PINK1*, a familial PD disease gene, and PINK1-dependent mitophagy (mitochondrial autophagy), the process of mitochondrial quality control that is disrupted by *PINK1* variants to cause PD [52]. Given that colocalization supports two distinct causal variants at this locus and that the nearest gene to a lead variant has a high prior probability of causality [53], the most parsimonious explanation is that *SETD1A* is a causal gene for PD, while *KAT8* is a causal gene for AD. However, the experimental evidence for *SETD1A* and *KAT8* is relatively weak compared to many of the other loci discussed in this paper, and this locus is especially gene-dense (GENCODE [54] Release 39 lists 49 protein-coding genes within 500 kilobases of rs11150601 and/or rs889555) so many other genes could be causal (we note that 500 kilobases is a somewhat arbitrary threshold; GWAS variants may regulate genes even greater distances away).

The next AD- and PD-associated variant was the ADRD lead variant rs450674 (chr16:79,574,511), located approximately 11 kilobases downstream of the transcription factor *MAF*. rs450674 had the same direction and magnitude of association with both disorders (ADRD OR = 1.04, *p* = 3.2 × 10^−8^; PD OR = 1.04, *p* = 2.1 × 10^−4^), and colocalization supported a shared causal variant (80.5% chance). Besides *MAF*, the only other coding gene within 500 kilobases is the oxidoreductase *WWOX*, involved in the regulation of apoptosis. Supporting the relevance of *WWOX* to AD, *WWOX* downregulation induced tau hyperphosphorylation in vitro [55], and Wwox knockout mice exhibited large increases in tau aggregation by 3 weeks of age [56] (though Wwox knockout may not be a realistic model for the much gentler perturbation of WWOX expression likely to be effected by a common GWAS variant). Supporting its relevance to PD, WWOX phosphorylation contributed to neuronal apoptosis upon treatment with the dopaminergic neurotoxin 1-methyl-4-phenyl-pyridinium (MPP +), a model of PD [57].

The next variants associated with both ADRD and PD were the ADRD lead variant rs5848 (chr17:44,352,876) and the PD lead variant rs850738 (chr17:44,357,262). Both variants are at the *GRN* locus: rs5848 is located in the 3′ untranslated region (UTR) of *GRN*, while rs850738 is located approximately 4 kilobases away in an intron of the neighboring gene *FAM171A2*. Both rs5848 (ADRD OR = 1.07, *p* = 2.4 × 10^−20^; PD OR = 1.07, *p* = 1.8 × 10^−12^) and rs850738 (ADRD OR = 0.96, *p* = 3.0 × 10^−6^; PD OR = 0.93, *p* = 1.3 × 10^−11^) had concordant directions of effect on the two disorders. Colocalization analysis supported ADRD and PD having two distinct causal variants at this locus (74.2% chance). *GRN* (progranulin) loss-of-function mutations are responsible for about 5–20% of FTD cases [58], and *GRN* has been explored as a candidate gene therapy for FTD [59]. Progranulin is thought to affect neurodegeneration through its effects on lysosomal function [58, 60]. Progranulin insufficiency has downstream proinflammatory and anti-neurotrophic effects and makes neurons more vulnerable to hypoxia, oxidative stress, and excitotoxicity [61]. While *GRN* is a strong causal candidate at this locus, *FAM171A2* may also play a role as a regulator of *GRN*: *FAM171A2* overexpression reduced progranulin levels in endothelial cells, while knockdown increased progranulin levels [62].

Finally, two variants at the *MAPT* (tau) locus were associated with both ADRD and PD: the PD lead variant rs62053943 (chr17:45,666,837), and rs199526 (chr17:46,770,341), a linkage disequilibrium (LD) proxy of the ADRD lead variant rs199515 (chr17:46,779,275) with *r*^2^= 0.911 in TopLD. Both rs62053943 (ADRD OR = 0.95, *p* = 5.1 × 10^−6^; PD OR = 0.76, *p* = 3.6 × 10^−68^) and rs199526 (ADRD OR = 0.95, *p* = 1.7 × 10^−8^; PD OR = 0.81, *p* = 1.8 × 10^−65^) showed much stronger associations with PD than with AD, but with the same direction of effect. Colocalization analysis supported two distinct causal variants at this locus (81.6% chance). Tau is the obvious causal gene candidate at this locus: it is a hallmark of AD and also appears to be involved in the etiology of PD [63]. However, another strong causal gene candidate that should not be overlooked is *KANSL1* (KAT8 regulatory NSL complex subunit 1), a regulator of *KAT8* [64]. Like *KAT8*, *KANSL1* regulates *PINK1* and PINK1-dependent mitophagy [52]. *KANSL1* also regulates autophagy in general [65] and causes oxidative stress when deficient [66].

### Genetic risk loci shared between ADRD and ALS

Four lead variants were associated with both ADRD and ALS at a family-wise error rate of 5%, after Bonferroni correction for the number of lead variants for both disorders (Table 3, Fig. 2). To address the concern that ADRD is an imperfect proxy for AD, we confirmed that all four variants had nearly identical odds ratios for ADRD and AD. Three of the four were nominally significant (*p* < 0.05) for AD; the fifth (rs871269) had *p* = 0.068.

**Table 3:** Lead variants for ADRD or ALS associated with both disorders at a family-wise error rate of 5%. Odds ratios and *p*-values for AD (from Kunkle et al.) are also included as a sensitivity analysis. A1 = effect allele; A2 = non-effect allele; OR = odds ratio; PP_shared/distinct_ = posterior probability that the two disorders share the same causal variant/have two distinct causal variants at the locus according to colocalization analysis. For ADRD, odds ratios and *p*-values are from the full 788,989 participants where available (i.e., for ADRD lead variants) and from the 487,511 participants with genome-wide summary statistics otherwise (i.e., for the ALS lead variant)

Locus	Variant	A1/A2	Lead for	AD		ADRD		ALS		PP_shared_	PP_distinct_	Nearest gene	Most evidence for
				**OR**	***p*-value**	**OR**	***p*-value**	**OR**	***p*-value**				
1	rs871269	T/C	ADRD	0.97	0.068	0.96	8.7 × 10^−9^	0.95	6.8 × 10^−6^	0.9%	30.1%	*TNIP1*	*GPX3*
2	rs785129	T/C	ADRD	1.04	0.017	1.04	2.4 × 10^−9^	1.05	4.3 × 10^−5^	12.0%	84.0%	*HS3ST5*	*HS3ST5*, *HDAC2*, *MARCKS*
3	rs199515	C/G	ADRD	1.05	0.011	1.06	9.3 × 10^−13^	1.05	1.2 × 10^−4^	26.0%	63.9%	*WNT3*	*MAPT*, *KANSL1*
4	rs2526377	A/G	ADRD	1.07	2.0 × 10^−6^	1.05	1.6 × 10^−12^	1.04	1.0 × 10^−4^	73.9%	5.9%	*TSPOAP1*	*TSPOAP1*

The first variant associated with both ADRD and ALS was the ADRD lead variant rs871269 (chr5:151,052,827), located in an intron of *TNIP1* and associated with reduced risk of both ADRD (OR = 0.96, *p* = 8.7 × 10^−9^) and ALS (OR = 0.95, *p* = 6.8 × 10^−6^). ALS also had a lead variant at this locus, rs10463311 (chr5:151,031,274), 21.5 kilobases away from the ADRD lead variant, but it was not associated with ADRD (OR = 1.01, *p* = 0.50). Colocalization analysis suggested that ADRD and ALS were unlikely to share a causal variant at this locus (0.9% chance). *TNIP1* (tumor necrosis factor alpha-induced protein 3) is a plausible causal gene at this locus because of its role in innate immunity and because TNIP1 has a protein–protein interaction with the protein product of the familial ALS gene *OPTN* [67]. Evidence is stronger for the second-nearest gene, *GPX3* (glutathione peroxidase 3), particularly for ALS. GPX3 forms a protein–protein interaction with another antioxidant enzyme, SOD1 (superoxide dismutase) [67], encoded by the first-discovered ALS risk gene [68]; GPX3 is downregulated in both ALS patients and ALS mouse models [69, 70]; and knockdown of *gpx3*, but not *tnip1*, caused motor defects in zebrafish [70]. Besides *TNIP1* and *GPX3*, the seven other protein-coding genes within 500 kilobases of rs871269 are *ANXA6* (annexin A6), the poorly characterized gene *CCDC69*, the cadherin family member *FAT2*, the glycolipid transporter *GM2A*, and the proton-coupled amino acid transporters *SLC36A1*, *SLC36A2*, and *SLC36A3*. Annexin A6 interacts with tau and may affect its subcellular localization within neurons [71], while mutations in GM2A cause an autosomal recessive neurodegenerative syndrome similar to Tay-Sachs disease [72].

The second variant associated with both ADRD and ALS was the ADRD lead variant rs785129 (chr6:114,291,731), located in the first intron of *HS3ST5* and associated with increased risk of both disorders (ADRD OR = 1.04, *p* = 2.4 × 10^−9^; ALS OR = 1.05, *p* = 4.3 × 10^−5^). Colocalization analysis supported two distinct causal variants at this locus (84.0% chance). HS3ST5 is one of seven heparan sulfate 3-O-sulfotransferase enzymes, which catalyze the addition of sulfate groups at the 3-OH position (“3-O-sulfation”) of glucosamine subunits within heparan sulfate disaccharide chains. A second member of the same family, *HS3ST1*, was also genome-wide significant for ADRD (lead variant rs6846529, chr4:11,023,507, OR = 1.07, *p* = 2.2 × 10^−17^). Heparan sulfates play a central role in protein aggregation by stabilizing aggregates, shielding them from proteolysis, and acting as cell-surface receptors to enhance the cellular uptake of aggregates [73]. Heparan sulfate sulfation patterns strongly affect rates of cellular uptake of tau, amyloid-beta, and alpha-synuclein aggregates, potentially contributing to the propagation of a variety of neuropathologies [74]. 3-O sulfation, in particular, has been shown to increase the cellular uptake of tau [75]. Another strong causal gene candidate at this locus is the second nearest gene, the histone deacetylase HDAC2, which represses genes involved in memory formation and synaptic plasticity (Guan et al. [76]). *Hdac2* knockdown restores cognition in a mouse model of AD [77] and histone deacetylase inhibitors have been proposed as neurodegenerative disease therapeutics [78, 79]. Besides HS3ST5 and *HDAC2*, the only other protein-coding gene within 500 kilobases of rs785129 is *MARCKS* (myristoylated alanine-rich C-kinase substrate), hyperphosphorylation of which appears to induce Yes-associated protein (YAP)-dependent necrosis of neurons in early-stage AD [80–82]. A monoclonal antibody against *MARCKS*’s upstream regulator HMGB1 strongly inhibited neurite degeneration and fully restored impaired cognition in an AD mouse model [81]. *MARCKS* is also a marker of neurite degeneration in mouse models of early-stage PD/dementia with Lewy bodies [83], suggesting transdiagnostic effects across neurodegenerative disorders. This locus is unusual in having three highly plausible causal gene candidates, and it is conceivable that the ADRD and ALS causal variants at this locus act through distinct causal genes (or through multiple causal genes, but to differing degrees), especially considering that colocalization supported distinct causal variants for the two disorders.

The third variant associated with both ADRD and ALS was the ADRD lead variant rs199515 (chr17:46,779,275), located in the first intron of *WNT3* at the *MAPT* locus. The effect size of this association was similar for ADRD (OR = 1.06, *p* = 9.3 × 10^−13^) and ALS (OR = 1.05, *p* = 1.2 × 10^−4^). Just like for ADRD and PD, colocalization analysis supported two distinct causal variants at this locus (63.9% chance). While the role of tau in ALS is less well-established than for AD and PD, recent research has shown that a specific phosphorylated tau species, pTau-S396, is mislocalized to synapses (rather than the cytosol) in motor cortex neurons from postmortem brains across ALS subtypes [84], and also more abundant in both postmortem motor cortex and cerebrospinal fluid compared to controls [85]. QC-01–175, a compound that selectively degrades tau, reversed mitochondrial fragmentation and oxidative stress in an in vitro model of ALS [84]. *KANSL1*, mentioned above in the context of PD, is also a plausible causal gene candidate for ALS, given that KANSL1 deficiency leads to oxidative stress via a SOD1-dependent mechanism [66]*.*

The final variant associated with both ADRD and ALS was the ADRD lead variant rs2526377 (chr17:58,332,680), located approximately 4 kilobases upstream from *TSPOAP1* and associated with increased risk of both ADRD (OR = 1.05, *p* = 1.6 × 10^−12^) and ALS (OR = 1.05, *p* = 3.4 × 10^−5^). Colocalization analysis supported a shared causal variant at this locus (73.9% chance). *TSPOAP1* encodes TSPO-associated protein 1, so named because it specifically interacts with translocator protein (TSPO) [86]. TSPO is a mitochondrial transmembrane protein that transports (translocates) cholesterol into mitochondria, which is the rate-limiting step in steroid synthesis [87]. TSPO positron emission tomography (PET) is widely used as a measure of microglial activation [88], and TSPO ligands reduce neuroinflammation and gliosis [87] and protect against neuropathology in mouse models of AD [89] and tauopathy [90]. *TSPOAP1*’s close relationship with TSPO makes it a strong causal gene candidate at this locus.

### Genetic risk loci shared between PD and ALS

Three lead variants at two loci were associated with both PD and ALS at a family-wise error rate of 5%, after Bonferroni correction for the number of lead variants for both disorders (Table 4, Fig. 2).

**Table 4:** Lead variants for PD or ALS associated with both disorders at a family-wise error rate of 5%. A1 = effect allele; A2 = non-effect allele; OR = odds ratio; PP_shared/distinct_ = posterior probability that the two disorders share the same causal variant/have two distinct causal variants at the locus according to colocalization analysis

Locus	Variant	A1/A2	Lead for	PD		ALS		PP_shared_	PP_distinct_	Nearest gene	Most evidence for
				**OR**	***p*-value**	**OR**	***p*-value**				
1	rs873786	T/C	PD	0.84	1.8 × 10^−21^	0.89	1.3 × 10^−7^	98.3%	1.6%	*GAK*	*GAK*, *TMEM175*
1	rs34311866	T/C	PD	0.81	1.0 × 10^−69^	0.93	1.1 × 10^−6^	98.3%	1.6%	*TMEM175*	*GAK, TMEM175*
2	rs62333164	A/G	Both	0.94	2.0 × 10^−10^	1.07	6.9 × 10^−9^	98.9%	1.1%	*CLCN3*	*NEK1*

The first two variants associated with PD and ALS were rs873786 (chr4:931,588) and rs34311866 (chr4:958,159), which the authors of the PD GWAS deemed to be two independent lead variants at the same locus according to conditional and joint analysis (COJO). rs873786 was associated with decreased risk of both PD and ALS (PD OR = 0.84, *p* = 1.8 × 10^−21^; ALS OR = 0.89, *p* = 1.3 × 10^−7^), as was rs34311866 (PD OR = 0.81, *p* = 1.0 × 10^−69^; ALS OR = 0.93, *p* = 1.1 × 10^−6^). Colocalization analysis suggested that PD and ALS were very likely to share a causal variant at this locus (98.3% chance); note that coloc does not consider the possibility that the same disorder might have multiple distinct causal variants at a locus. rs873786 is an intronic variant in *GAK*, while rs34311866 is a missense variant (p.M393T) in the neighboring gene *TMEM175*. Both *GAK* and *TMEM175* are strong causal gene candidates at this locus. GAK has been reported to form an autophagy-related protein complex with LRRK2 (a Mendelian PD disease gene and PD GWAS hit), RAB29 (another PD GWAS hit), HSPA8, and BAG5 [91]. Knockdown of the Drosophila homolog of GAK, auxilin (aux), in dopaminergic neurons led to dopaminergic neuron loss and parkinsonian-like symptoms in fruit flies [92]; similar effects were observed in mice lacking microglial *GAK* and fruit flies lacking glial aux, apparently mediated by disruption to glial autophagy [93]. Meanwhile, TMEM175 deficiency in cultured neurons led to unstable lysosomal pH and consequently impaired lysosomal activity, autophagy, glucocerebrosidase activity, and mitochondrial respiration as well as increasing alpha-synuclein aggregation when the neurons were seeded with alpha-synuclein fibrils [94]. A follow-up study by the same authors showed that rs34311866/p.M393T resulted in many of these same phenotypes, albeit to a lesser extent than a full knockout of *TMEM175* [95]. Given that rs873786 and rs34311866 are independent association signals, it is quite plausible that both *GAK* and *TMEM175* are causal genes for both disorders, with rs873786 acting through *GAK* and rs34311866 (the *TMEM175* missense variant) acting through *TMEM175*.

The final variant associated with both PD and ALS was rs62333164 (chr4:169,662,006), a lead variant for both disorders that had opposite directions of association with PD (OR = 0.94, *p* = 2.0 × 10^−10^) and ALS (OR = 1.07, *p* = 6.9 × 10^−9^). Colocalization analysis did not find strong evidence for either a shared causal variant (33.8% chance) or two distinct causal variants (24.0% chance). rs62333164 is an intronic variant in *CLCN3*, a voltage-gated chloride and proton channel with unclear relevance to neurodegeneration. However, the next-nearest gene, *NEK1*, is a strong causal gene candidate at this locus: *NEK1* loss-of-function variants are present in ~ 3% of ALS cases and associated with 8.2-fold increased odds of ALS [96]. NEK1 is involved in the DNA damage response, like several other familial ALS-associated genes [97], and ALS patient-derived motor neurons carrying *NEK1* loss-of-function mutations displayed increased DNA damage and an impaired DNA damage response [98]. NEK1 deficiency also promotes RIPK1-dependent apoptosis and necroptosis of endothelial cells, leading to disrupted blood–brain barrier integrity; supporting the relevance of this mechanism to PD, RIPK1 inhibition reduces neuroinflammation and alpha-synuclein aggregation in the brains of NEK1-deficient mice [99]. The discordant directions of rs62333164’s associations with the two disorders suggest the possibility of multiple causal genes. The remaining protein-coding genes within 500 kilobases of rs62333164 are *AADAT*, a mitochondrial transaminase; *HPF1*, a histone parylation factor that like *NEK1* is involved in the DNA damage response; *MFAP3L*, a kinase involved in cell proliferation and metastasis; and *SH3RF1*, a ubiquitin ligase.

## Discussion

Although GWAS have indicated a certain degree of genetic overlap between AD, PD, and ALS, the specific genetic variants underlying this overlap have remained largely elusive. In this study, we expand the landscape of variants associated with multiple of these three neurodegenerative disorders. Specifically, we find that eleven loci with GWAS hits for one disorder are also associated with one or both of the other disorders, at a threshold equivalent (in terms of family-wise error rate control) to genome-wide significance. This stringency is a key strength of our approach, relative to other approaches to pleiotropic locus discovery.

Of the eleven loci, one was associated with all three disorders (near *MAPT*/*KANSL1*), five with ADRD and PD (near *LCORL*, *CLU*, *SETD1A*/*KAT8*, *WWOX*, and *GRN*), three with ADRD and ALS (near *GPX3*, *HS3ST5*/*HDAC2*/*MARCKS*, and *TSPOAP1*), and two with PD and ALS (near *GAK*/*TMEM175* and *NEK1*). Three of these loci contain genes implicated in neurodegenerative disorders via loss-of-function variation (*CLU* for AD, *GRN* for FTD, *NEK1* for ALS), consistent with the known phenomenon that GWAS hits tend to occur near Mendelian disorder genes for similar disorders [100]. At least three of the eleven loci harbor genes (*GAK*/*TMEM175*, *GRN*, *KANSL1*) that may affect neurodegeneration via effects on lysosomal function or autophagy, one (*TSPOAP1*) via neuroinflammation and immunity, two (*GPX3*, *KANSL1*) via the oxidative stress response, and another (*NEK1*) via the DNA damage response. Thus, our associations support lysosomal/autophagic dysfunction, neuroinflammation, adaptive immunity, oxidative stress, and the DNA damage response as transdiagnostic processes underlying multiple neurodegenerative disorders. Notably, despite extensive pleiotropy at the *APOE* locus with cardiometabolic and other traits [101], rs429358 (which differentiates APOE4 from APOE3) was not significantly associated with either PD (OR = 1.01, *p* = 0.46) or ALS (OR = 0.98, *p* = 0.31).

Colocalization analysis supported a shared causal variant between ADRD and PD at the *CLU* (88.4% chance), *WWOX* (80.4% chance), and *LCORL* (65.3% chance) loci; between ADRD and ALS at the *TSPOAP1* locus (73.9% chance); and between PD and ALS at the *NEK1* (98.9% chance) and *GAK*/*TMEM175* (98.3% chance) loci. Conversely, colocalization supported distinct causal variants for ADRD and PD at the *SETD1A*/*KAT8* (100.0% chance), *MAPT* (81.6% chance), and *GRN* (74.2% chance) loci and for ADRD and ALS at the *HS3ST5*/*HDAC2*/*MARCKS* (84.0% chance) and *MAPT* (63.9% chance) loci. We note that sharing a causal variant is not at all the same as sharing a causal gene, since the same GWAS variants often regulate multiple distinct genes, many of which may not have any causal relationship to the trait [102, 103], and multiple distinct variants may regulate the same gene.

Two of the eleven loci were associated with increased risk of one neurodegenerative disorder but decreased risk of another: *LCORL* (specifically, rs34025766) with ADRD and PD, and *NEK1* (specifically, rs62333164) with PD and ALS. To our knowledge, this phenomenon (discordant directions of association of the same variant with two different neurodegenerative disorders) has only been reported once previously, for APOE4 in AD versus age-related macular degeneration [104]. These loci are particularly interesting candidates for experimental follow-up—especially the *NEK1* locus, where existing experimental evidence linking NEK1 deficiency to both PD and ALS fails to explain why rs62333164 has opposite directions of association with the two disorders.

This study has several limitations. First, our choice to use a stringent 5% family-wise error rate threshold may be overly conservative, and more shared loci could potentially have been discovered with a more relaxed threshold.

Second, our colocalization analysis assumes that each disorder has at most one causal variant per locus. This does not account for the possibility that the same locus may have multiple distinct causal variants for the same disorder. The coloc method we use for colocalization has recently been extended to account for this possibility [105], but this extension requires specifying the linkage disequilibrium matrix between all pairs of variants at the locus and is vulnerable to bias when this matrix is derived from a different cohort than the GWAS, which is unavoidable when using GWAS summary statistics for which the underlying individual-level data are not available.

Third, the AD, PD, and ALS GWAS participants generally lack gold standard pathology-based diagnoses, and some may have pathologies that do not correspond to their diagnosis, or mixed pathologies. In particular, as discussed above, the AD GWAS includes “proxy cases” from the UK Biobank who self-reported having a parent or sibling with AD or dementia, and some of these parents or siblings may have had frontotemporal dementia, vascular dementia, or dementia with Lewy bodies rather than AD [106]. Fortunately, all of the variants associated with both ADRD and another disorder had similar odds ratios for AD as for ADRD, and all but one were nominally significant (*p* < 0.05) for AD, suggesting that the inclusion of related dementias in the ADRD case definition is not leading to false positives. A related issue is that our ADRD GWAS lumps together APOE4^+^ and APOE4^−^ participants, even though APOE4-related AD may have a distinct etiology and be less influenced by genetic variants other than APOE4 [107].

Fourth, shared controls between the original GWAS will tend to inflate the degree of pleiotropy reported here. To our knowledge, the only two instances of shared controls arise from the UK Biobank, used in the ADRD and PD GWAS (up to 338,440 shared UK Biobank controls, since the ADRD GWAS used 338,440 UK Biobank controls, and the PD GWAS used 436,419), and the Wellcome Trust Case Control Consortium (WTCCC) [108], used in the PD and ALS GWAS (5200 shared controls). While the WTCCC shared controls are a small percentage of the total controls in each study (0.4% of the PD GWAS’s 1,417,791 controls and 4.2% of the ALS GWAS’s 122,656 controls), the shared UK Biobank controls are a large percentage of the total controls (up to 49.9% of the ADRD GWAS’s 677,663 controls and 23.9% of the PD GWAS’s 1,417,791 controls). Fortunately, the AD GWAS we used for our sensitivity analysis [30] did not include any UK Biobank participants, and all our ADRD results were consistent in this AD GWAS, indicating that the shared UK Biobank controls do not noticeably skew the results.

Finally, assigning causal genes to GWAS loci is a notoriously difficult and unsolved problem [109]. Our hypotheses about causal gene candidates should not be treated as definitive.

## Conclusion

In sum, we identify eleven genetic risk loci shared between two or more of AD, PD, and ALS. These loci support lysosomal/autophagic dysfunction (*GAK*/*TMEM175*, *GRN*, *KANSL1*), neuroinflammation and immunity (*TSPOAP1*), oxidative stress (*GPX3*, *KANSL1*), and the DNA damage response (*NEK1*) as transdiagnostic processes underlying multiple neurodegenerative disorders.

## Data Availability

See the “Methods” section.

## Abbreviations

GWAS: Genome-wide association studies
AD: Alzheimer’s disease
ADRD: Alzheimer’s disease and related dementias
PD: Parkinson’s disease
ALS: Amyotrophic lateral sclerosis
